# Loss of CD44 re-educates pancreatic cancer-associated fibroblasts modulating their fibrotic and immunosuppressive functions

**DOI:** 10.1038/s41419-026-09155-5

**Published:** 2026-08-15

**Authors:** Sven Máté Treffert, Yvonne Madeleine Heneka, Julie Martin, Alessandra Gargano, Larissa Launhardt, Özlem Akilli, Eliana Stanganello, Geoffroy Andrieux, Dominique Hoch, Steffen Joachim Sonnentag, Lisa-Marie Mehner, Leonel Munoz-Sagredo, Michelle Christ, Véronique Orian-Rousseau

**Affiliations:** 1 Karlsruhe Institute of Technology, Institute of Biological and Chemical Systems, Functional Molecular Systems (IBCS-FMS), Kaiserstraße 12, Karlsruhe, 76131 Germany; 2 TRON Translational Oncology at the University Medical Center, JGU-Mainz, Mainz, 55131 Germany; 3 Institute of Medical Bioinformatics and Systems Medicine, Medical Center, University of Freiburg, Faculty of Medicine, University of Freiburg, Freiburg, 79085 Germany; 4 Faculty of Medicine, Universidad de Valparaiso, Anglos 655, 2540064 Vina del Mar, Chile

**Keywords:** Cancer microenvironment, Tumour immunology

## Abstract

Pancreatic tumors are characterized by a prominent desmoplastic stroma that can account for up to 90% of the tumor. Given the marked upregulation of CD44, a family of transmembrane glycoproteins, in pancreatic cancer-associated fibroblasts (CAFs), we investigated its role in regulating myofibroblastic and inflammatory CAF phenotypes. Conditional deletion of *Cd44* in fibroblastic cells in *Cd44*^*fl/fl*^*;PdgfrβCreER*^*T2*^ mice significantly reduced tumor growth and was associated with a significant reduction in intratumoral regulatory T cells (T_reg_). Consistently, in human CAFs CRISPR/Cas9-edited to delete *CD44*, the fibroblasts’ morphology changed drastically: CAFs lost their elongated phenotype and adopted a round shape, reflecting their inactivation. This was accompanied by a significant downregulation of activation markers, unresponsiveness to exogenous stimuli, and reduced contractile activity. Furthermore, CD44 ablation decreased extracellular matrix production and altered the immunomodulatory cytokine secretion, highlighting its role in both fibrosis and immune regulation. Finally, CD44-deficient CAFs exhibited a reduced capacity to suppress anti-tumor immune responses by failing to induce immunosuppressive programs in dendritic cells (DCs) and by increasing cytotoxic T cell (CTL)-mediated tumor cell killing. Collectively, we identify CD44 as a regulator of CAF activation and immunosuppressive function, linking stromal remodeling to immune evasion in pancreatic cancer and supporting CD44 as a potential therapeutic strategy to reprogram the tumor stroma and enhance anti-tumor immunity.

## Introduction

Pancreatic ductal adenocarcinoma (PDAC) is the most common form of pancreatic cancer [1]. As many patients present with systemic and non-resectable disease, the overall five-year survival rate is low (around 10%) [2]. One hallmark is the prominent desmoplastic stroma, which can represent up to 90% of the total tumor volume [3] and is composed of extracellular matrix (ECM), immune cells, endothelial cells, and cancer-associated fibroblasts (CAFs). Transcriptional analysis of activated pancreatic stellate cells (PSCs) has revealed an upregulation of extracellular matrix, remodeling enzymes, growth factors, and other signatures characteristic of CAFs [4]. This supports the notion that PSCs can differentiate into CAFs during PDAC progression [5]. Notably, CAFs represent a highly heterogeneous population comprising multiple subpopulations, which arise not only from PSCs but also from bone marrow-derived mesenchymal cells and from epithelial or endothelial cells [6–9].

Two of the main subclusters are defined as inflammatory (iCAF) and myofibroblastic (myCAF) CAFs. iCAFs are characterized by low levels of ɑ smooth muscle actin (ɑSMA) and high levels of pro-inflammatory cytokines like IL-6, CXCL12, and CCL2 [10]. They are mainly induced through the IL-1/Lif/JAK-STAT and the TNF-ɑ pathways, both converging on NF-$\kappa {\rm{B}}$ downstream [11]. myCAFs exhibit high ɑSMA expression and produce abundant ECM with an anisotropic arrangement of extracellular matrix proteins, thus acting as a physical barrier impairing immune cell infiltration and drug delivery. Their most potent inducer is transforming growth factor β1 (TGFβ1) [11].

By secreting cytokines, chemokines and growth factors, CAFs affect immunosuppressive cells like tumor-associated macrophages (TAMs) [12], myeloid-derived suppressor cells (MDSCs) [13], dendritic cells (DCs) [14], and ultimately T lymphocytes. Several studies have shown that CAFs can modulate the identity and function of T cells, thus hindering CD8^+^ cytotoxic T cell (CTL)-mediated responses against the tumor as well as favoring the regulatory T cells (T_reg_) phenotype [15, 16]. This abundance of immunosuppressive cell types (MDSCs, TAMs, T_reg_), as well as the dampened activity of effector immune cells (CD4^+^ and CD8^+^ T cells), renders pancreatic cancer extremely non-immunogenic and non-responsive to immunotherapy [17].

Given their abundance, plasticity, and impact on disease progression, CAFs represent a compelling therapeutic target. However, depleting studies of CAFs in mouse models of pancreatic cancer generated contradictory results. Many studies found that the depletion of CAFs worsened the outcome, resulting in poorly differentiated and more aggressive tumors [18, 19]. Others showed that, depending on the affected population, targeting various subtypes in the distinct subpopulations resulted in tumor-promoting or tumor-restricting outcomes [20]. Therefore, CAF-targeting strategies have been pursued with increasing caution and shifted toward the reprogramming of CAFs into a less tumor-promoting state [21].

Our group previously showed that blocking the CD44 isoform, CD44v6, in pancreatic orthotopic xenograft and the autochthonous *Kras*^*G12D/+*^*;Trp53*^*R172H/+*^*;Pdx1Cre* (KPC) mouse models, reduced primary tumor volume and metastatic burden, even if the species-specific blocking peptides only targeted the stromal CD44v6 [22, 23]. This suggested that CD44 expression in stromal cells critically influences pancreatic tumor progression. However, the mechanistic contribution of CD44 to CAF activation, desmoplastic stroma formation, and immunoregulatory functions in PDAC remains unclear. Here, we demonstrate that CD44 is essential for the maintenance of an activated and immunosuppressive CAF phenotype in PDAC, supporting its potential as a target for stroma-reprogramming strategies.

## Results

### *Cd44* deletion in PDGFRβ^+^ stromal cells is associated with reduced primary tumor volume

**Fig. 1:**
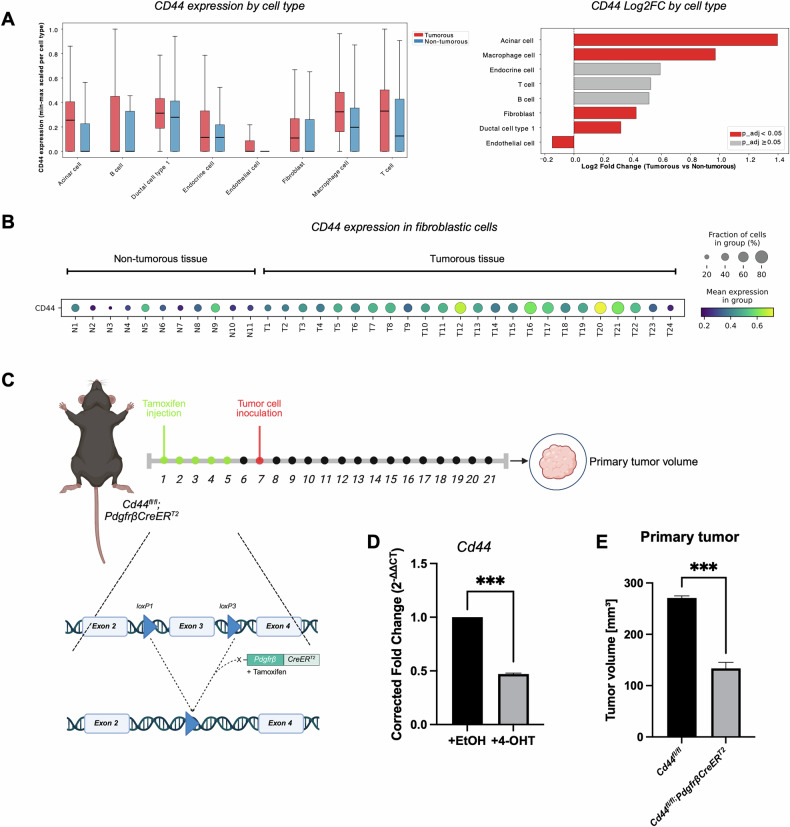
Expression of *CD44* in tumor-associated stromal cells and its importance in PDGFRβ^+^ fibroblastic cells during primary tumor progression. **A** Publicly available human pancreatic single-cell RNA-seq datasets were analyzed to compare *CD44* expression between non-tumorous and tumorous tissues across annotated cell types. Left, boxplots showing min-max scaled *CD44* expression within each cell type. Right, bar plot showing the corresponding log2 fold change in *CD44* expression between tumorous and non-tumorous tissues. Statistical significance was assessed using the Wilcoxon rank-sum test (*p*-value < 0.05). Cell-type annotations were adopted from the original publication. **B** Dot plot showing *CD44* expression in fibroblastic cells across non-tumorous (N1-N11) and tumorous (T1-T24) pancreatic tissue samples. Color intensity indicates mean *CD44* expression per sample, whereas dot size represents the proportion of fibroblastic cells expressing *CD44*. **C** Experimental scheme of the *Cd44*^*fl/fl*^*;PdgfrβCreER*^*T2*^ mice treated with tamoxifen to induce the *Cd44* knockout in PDGFRβ^*+*^ cells and orthotopic tumor cell inoculation of FC1245 cells. **D** Primary pancreatic fibroblasts were isolated from *Cd44*^*fl/fl*^*;PdgfrβCreER*^*T2*^ mice and treated with either 25 µM 4-OHT or ethanol as control. *Cd44* expression was analyzed at day seven by qPCR. *Gapdh* and *βactin* were used as reference genes. Data are means ± S.E. Statistical significance was determined using the one sample *t* test. *n* = 3. ***p value < 0.001. **E** Primary tumor volume of tamoxifen-treated *Cd44*^*fl/fl*^*;PdgfrβCreER*^*T2*^ and corresponding control animals. Data are means ± S.E. Statistical significance was determined using the unpaired one-sided *t* test. *N* = 3. ***p value < 0.001. Parts of the Figure: Created in BioRender. Treffert, S. (2026) https://BioRender.com/fxdd023, Treffert, S. (2026) https://BioRender.com/efu5df8.

Previous studies by our group highlighted the role of stromal CD44 during pancreatic tumor progression [22, 23]. To identify which stromal compartment contributes to this effect, we analyzed publicly available single-cell RNA sequencing (scRNAseq) data from pancreatic cancer patients [24]. Comparative analysis of non-tumorous and tumorous pancreatic tissue (Fig. 1A) revealed a broad increase in CD44 expression across multiple cellular compartments, including acinar cells, ductal cells, endothelial cells, macrophages, and fibroblastic cells. Among these, fibroblastic cells displayed a significant upregulation of CD44 expression in tumor samples (Fig. 1B). This enrichment suggests a prominent association of CD44 with CAFs in the PDAC stroma.

To investigate the role of CD44 expression in vivo, we crossed *Cd44*^*fl/fl*^ mice with mice expressing an inducible Cre recombinase (CreER^T2^) under the control of the *Pdgfrβ* promoter (Fig. 1C) [25, 26]. This enables the tamoxifen-inducible deletion of *Cd44* in PDGFRβ*-*expressing stromal cells*,* including pericytes, fibroblasts, PSCs and CAFs. Primary fibroblastic cells isolated from the pancreas of *Cd44*^*fl/fl*^*;PdgfrβCreER*^*T2*^ mice, characterized by ɑSMA and PDGFRβ expression (Fig. S1A), showed significantly reduced *Cd44* expression following 4-Hydroxytamoxifen (4-OHT) treatment compared to ethanol-treated controls (Fig. 1D). Experimental mice were injected intraperitoneally with tamoxifen (TAM) for 5 days followed by the orthotopic injection of FC1245 cells from the KPC model. Strikingly, *Cd44* deletion in PDGFRβ^+^ stromal cells reduced primary tumor volume by approximatively 50% two weeks after cancer cell implantation compared to control mice (Fig. 1E).

### CD44 expression in PSCs supports their TGFβ1-mediated activation into a CAF-like state

**Fig. 2:**
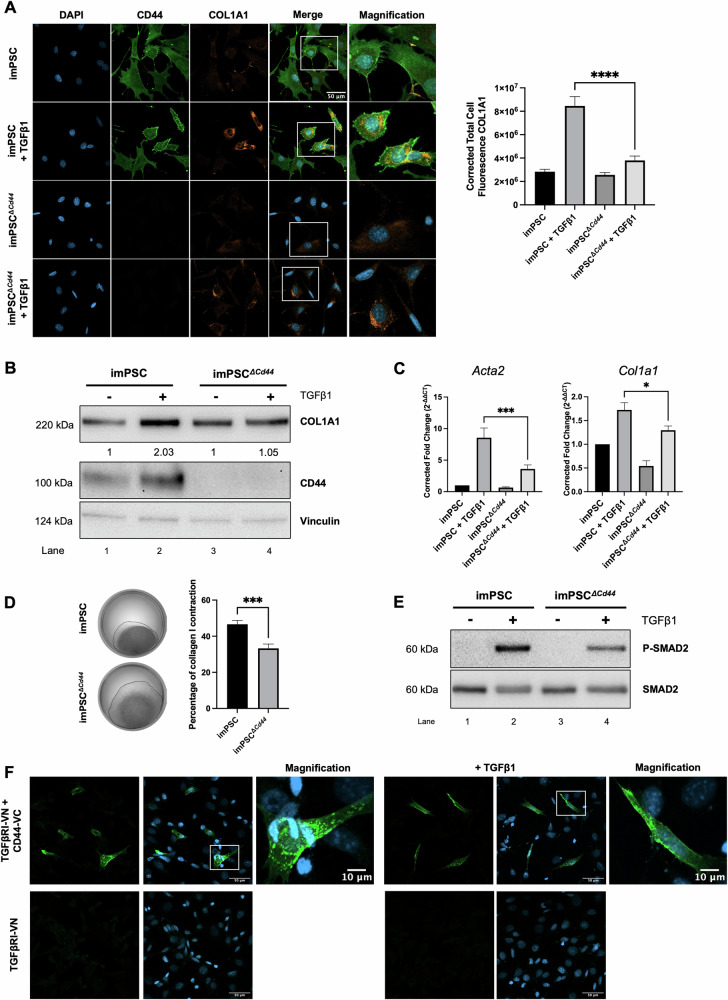
Knockout of *Cd44* in pancreatic stellate cells impairs the induction of a CAF-like state. **A** IF analysis of imPSCs and imPSC^*ΔCd44*^ after induction with recombinant TGFβ1 (50 ng/ml) stained for CD44 (AlexaFluor488, green), COL1A1 (AlexaFluor546, orange); nuclei were counterstained with DAPI (blue). Scale bar, 50 µm. Corrected total cell fluorescence was calculated with ImageJ. *N* = 3, *n* = 30 pictures per condition. Data are means ± S.E. Statistical significance was determined using the one-sided unpaired Student’s *t* test. ****p value < 0.0001. **B** Analysis of CD44 and COL1A1 on protein level after treatment of imPSCs and imPSC^*ΔCd44*^ with TGFβ1 by western blot. Vinculin served as loading control. **C** Gene expression of *Acta2* and *Col1a1* of TGFβ1-induced imPSCs and imPSC^*ΔCd44*^ was analyzed by qPCR. *Gapdh* and *βactin* were used as reference genes. *N* = 3 Data are means ± S.E. One-way ANOVA and Holm-Šídák’s multiple comparisons post-hoc test were used for statistical analysis. *p value < 0.05; ***p value < 0.001. **D** imPSCs and imPSC^*ΔCd44*^ were seeded into a 3D type I collagen matrix and cultured for 48 h. The percentage of contraction was calculated by measuring the area of the contracted gel. *N* = 3. Data are means ± S.E. Statistical significance was determined using the one-sided unpaired Student’s *t* test. ***p value < 0.001. **E** imPSCs and imPSC^*ΔCd44*^ treated 30 min with recombinant TGFβ1 (50 ng/ml) were subjected to western blot analysis for P-SMAD2 and SMAD2. **F** BiFC analysis of the association between CD44 and TGFβRI in imPSCs. imPSCs were transfected with TGFBRI-VN and CD44-VC fusion constructs. Reconstitution of the fluorescent protein generated a fluorescence signal in co-transfected cells (green), indicating a close spatial association between CD44 and TGFβRI. Cells transfected with TGFBRI-VN alone served as negative controls and showed no detectable fluorescence signal. Nuclei were counterstained with DAPI (blue). Representative confocal images are shown. Scale bar, 50 µm.

The reduced tumor growth observed following *Cd44* deletion in PDGFRβ^+^ stromal cells prompted us to investigate the role of CD44 in PSCs, a well-known precursor population of CAFs in PDAC [4, 11]. To study the influence of CD44 on PSC activation toward a CAF-like state, we deleted *Cd44* using CRISPR/Cas9 in an immortalized murine pancreatic stellate cell line (imPSC) [27], abbreviated imPSC^*ΔCd44*^ (Fig. S1B). We assessed their activation by induction with TGFβ1 and measured the expression of myofibroblastic activation markers α-1 type I collagen (*Col1a1*) and ɑSMA (*Acta2*) (Fig. 2A–C). In striking contrast to imPSCs, imPSC^*ΔCd44*^ failed to upregulate these markers upon TGFβ1 induction. Immunofluorescence analysis revealed that COL1A1 expression more than doubled upon TGFβ1 induction in imPSCs, while the increase was significantly less in the imPSC^*ΔCd44*^ (Fig. 2A). In line, western blot analysis confirmed that TGFβ1 more than doubled COL1A1 levels in imPSCs (1.0 to 2.03), whereas they remained stable in imPSC^*ΔCd44*^ cells (Fig. 2B). Consistent results were obtained upon CD44 knockdown using siRNA (Fig. S2A). qPCR analysis after TGFβ1 treatment showed decreased *Col1a1* in the imPSC^*ΔCd44*^, while *Acta2* increased significantly less compared to imPSCs (Fig. 2C). We next assessed the ability of imPSCs and imPSC^*ΔCd44*^ to contract a 3D type I collagen gel [28, 29] (Fig. 2D). After 48 h of culture, imPSC^*ΔCd44*^ exhibited markedly reduced contractile activity compared to imPSCs.

To investigate whether CD44-dependent effects in PSC activation are due to CD44 being the receptor for HA, we analysed the expression of hyaluronan synthase genes (Has1, Has2, and Has3). No significant differences were observed between imPSCs and imPSC^*ΔCd44*^ (Fig. S2B), suggesting that CD44-mediated effects are not associated with increased HA abundance.

As CD44 proteins are co-receptors for a variety of cell surface receptors, we assessed whether the knockout of *Cd44* directly influences the TGFβ1-induced Smad pathway. We showed that the knockout of *Cd44* reduced phosphorylation of SMAD2 (P-SMAD2) in imPSC^*ΔCd44*^ (Fig. 2E). To further investigate the relationship between CD44 and TGFβRI, we performed bimolecular fluorescence complementation (BiFC) assays using TGFBRI-VN and CD44-VC fusion constructs. Co-expression of both constructs in imPSCs resulted in a clear fluorescence signal, whereas control cells expressing TGFBRI-VN alone showed no detectable fluorescence (Fig. 2F). These findings indicate a close spatial association between CD44 and TGFβRI in PSCs and support a role for CD44 in TGFβ1 signaling. Consistently, co-immunoprecipitation experiments revealed an association between CD44 and TGFβRI (Fig. S2C). It is reported that TGFβ1-mediated activation involves EMT-like processes [30]. qPCR analysis of TGFβ1-induced imPSCs showed that the knockout of *Cd44* led to a reduction of the EMT transcription factors *Snai1*, *Twist1*, and *Zeb1* in imPSC^*ΔCd44*^ and a higher expression of *EpCam* (Fig. S2D). Using vimentin-chromobody transfected cells, we confirmed reduced expression of this mesenchymal cytoskeletal protein [31] (Fig. S2E).

Together, we demonstrate that CD44 is a critical regulator for TGFβ1-driven PSC activation and their functional transition toward a CAF-like phenotype.

### CD44 in pancreatic fibroblastic cells induces tumor stroma-like parallel orientation of collagen I fibers

**Fig. 3:**
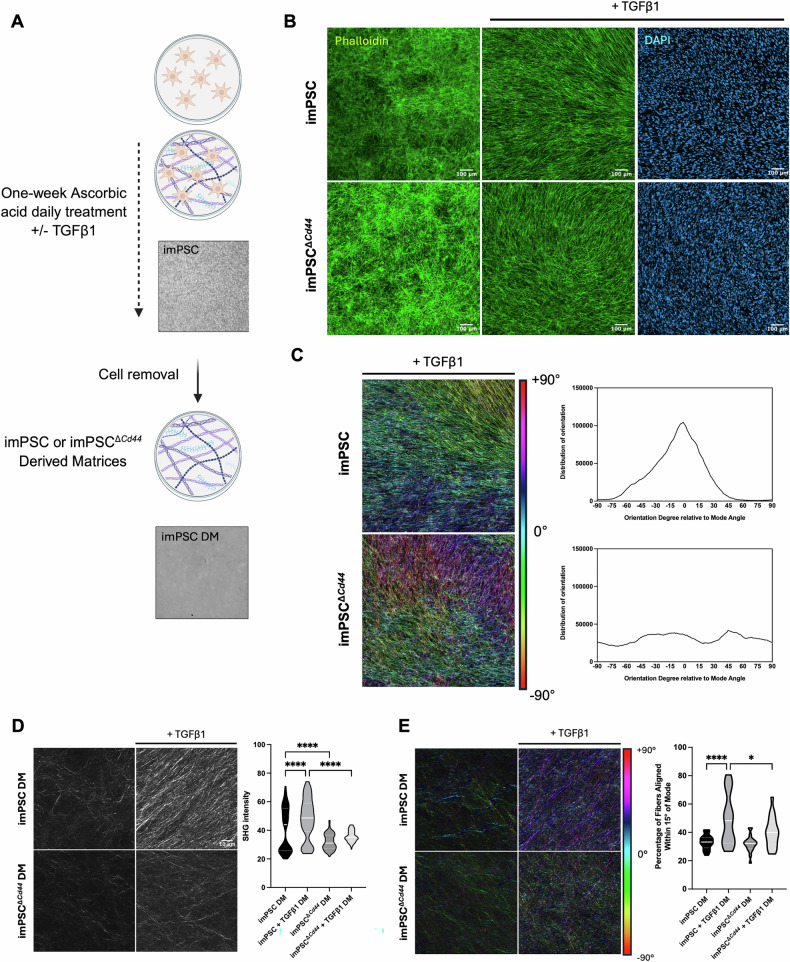
*Cd44* knockout in imPSCs impairs extracellular matrix production and organization. **A** Experimental strategy to generate matrices derived from imPSCs and imPSC^*ΔCd44*^. Schematic illustration created in BioRender. Martin, J. (2026) https://BioRender.com/gfwiiwf. **B** Representative images of imPSCs and imPSC^*ΔCd44*^ during ECM production with or without TGFβ1 stimulation prior to cell removal. Nuclei are stained with DAPI (blue) and Phalloidin in green. Scale bars, 100 µm. **C** F-actin fibers digitally pseudo-colored to depict cell orientation angles. The colored bar on the right indicates angle distributions normalized to mode angle (0°, cyan). Cell alignment was quantified by measuring fiber angle distribution. **D** Reconstituted confocal images of Second Harmonic Generation (SHG) microscopy of imPSC- and imPSC^*ΔCd44*^-derived matrices (DM). Quantification of SHG signal intensity reflects the amount of aligned collagen fibers. Scale bar, 10 µm. **E** Analysis of ECM fiber orientation from SHG images in D. Fiber alignment was quantified as the percentage of fibers oriented within 15° of the mode angle. Scale bar, 10 µm. Statistical significance was determined by one-way ANOVA and Holm-Šídák’s multiple comparisons post hoc test. *N* = 3, *n* = 5 pictures per sample. *p value < 0.05; ****p value < 0.0001.

Activated PSCs and CAFs are central to the production, organization and remodeling of the extracellular matrix across various tissue microenvironments. To test the capacity of imPSCs that express or do not express CD44 to control ECM deposition, we compared imPSC-/ imPSC^*ΔCd44*^-derived matrices (DM) [32] (Fig. 3A). These matrices recapitulate key biochemical and architectural features of the in vivo TME [33]. After generation of a PSC-DM, clear morphological differences became apparent between imPSC^*ΔCd44*^ and imPSCs following induction into a CAF-like state by TGFβ1 treatment. imPSCs expressing CD44 adopted an elongated spindle-like morphology (Fig. 3B) and organized into parallel patterns (Fig. 3C). Since tumor-associated ECM synthesized by CAFs typically displays a parallel organization [34], we then investigated whether CD44 expression influences the topography of imPSC-DM by assessing collagen alignment using second harmonic generation (SHG) analysis (Fig. 3D). We observed a significant reduction of organized collagen fibers in imPSC^*ΔCd44*^-DM compared to the DM of imPSCs, reflected in a significant reduction of the SHG intensity. In addition, the level of organization of the imPSC-produced collagen fibers was quantified by measuring the relative orientation angles of fibers [35]. The average percentage of parallel fibers that were oriented within 15° of the mode angle was determined with the OrientationJ plugin of Fiji. The measured percentages after TGFβ1 induction were 49% for the imPSC- and 32% for the imPSC^*ΔCd44*^-DM (Fig. 3E). With these results we show that CD44 is necessary for the organization of the ECM fibers produced by imPSCs.

### Removal of CD44 from human pancreatic CAFs drastically impacts their activation as reflected by a drastic change in morphology

**Fig. 4:**
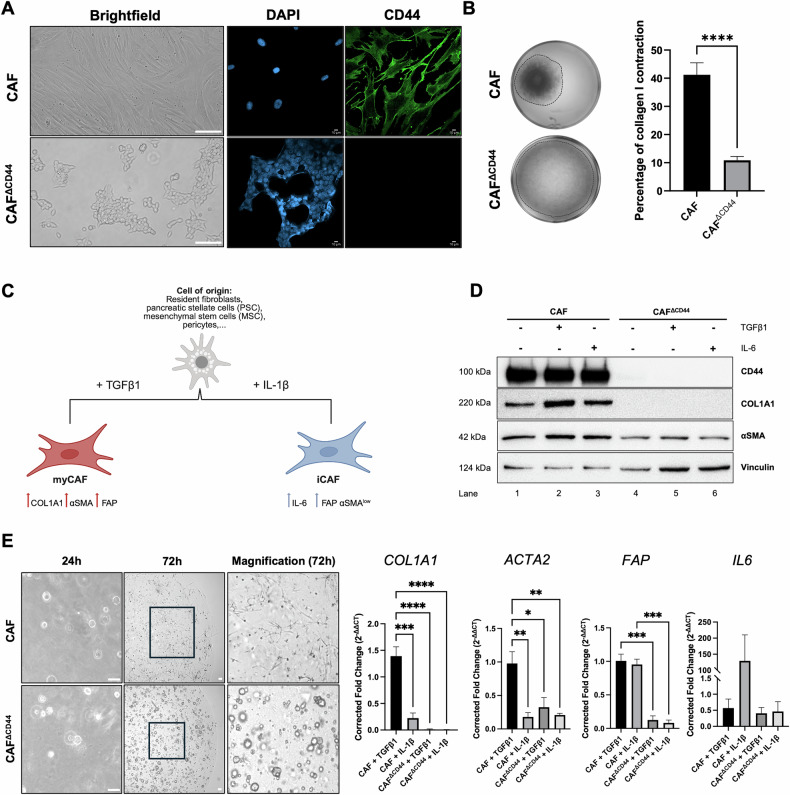
*CD44* knockout in human CAFs influences their morphology, activity and expression of CAF activation markers. **A** Representative image of the morphology of CAFs in comparison to CAF^*ΔCD44*^. Scale bar, 100 µm (left); 10 µm (right). IF staining against CD44 (AlexaFluor488, green) in CAFs and CAF^*ΔCD44*^. Nuclei were counterstained with DAPI. **B** CAFs and CAF^*ΔCD44*^ were seeded into 3D collagen I matrices, incubated for 48 h, and the percentage of contraction was calculated. *N* = 3. Data are means ± S.E. Statistical significance was determined using the unpaired one-sided Student’s *t* test. ****p value < 0.0001. **C** The two predominant CAF subtypes, myCAFs and iCAFs, are induced by either TGFβ1 or IL-1β and up- or downregulate their respective activation markers. Schematic illustration: Created in BioRender Treffert, S. (2026) https://BioRender.com/7t44o62. **D** CAFs and CAF^*ΔCD44*^ were treated with recombinant TGFβ1 (50 ng/ml) or IL-6 (20 ng/ml) for 24 h. Protein expression of CD44, COL1A1, and ɑSMA with Vinculin as loading control. **E** CAFs and CAF^*ΔCD44*^ were seeded in Matrigel domes and induced with recombinant TGFβ1 (50 ng/ml) or IL-1β (20 ng/ml) for 24 h. Representative images of the CAFs and CAF^*ΔCD44*^ morphology 24 h and 72 h after seeding. Scale bars, 50 µm. *COL1A1*, *ACTA2*, *FAP*, and *IL6* gene expression was analyzed by qPCR 24 h after induction (72 h after seeding). *GAPDH* was used as a reference gene. *N* = 3. Data are means ± S.E. One-way ANOVA and Holm-Šídák’s multiple comparisons post hoc test were used for statistical analysis. *p value < 0.05; **p value < 0.01; ***p value < 0.001; ****p value < 0.0001.

Our findings show that *Cd44* deletion in PSCs limited their transition into a myCAF-like state. To find out whether our findings were translatable to the human context, we examined CD44 function in human tumor-derived CAFs. Surprisingly, CRISPR/Cas9-edited CAFs, without *CD44* expression (Fig. S3A), lost their typical elongated spindle-like shape and exhibited a small and round morphology (Fig. 4A). This was not observed in scramble sgRNA-mCherry-transduced control cells (Fig. S3B). Because rounded fibroblasts are typically less active [36], we examined their ability to contract a 3D type I collagen gel. Interestingly, 48 h after embedment, CAF^*ΔCD44*^ contracted significantly less compared to *CD44*-expressing controls (Fig. 4B), in accordance with our results on imPSC^*ΔCd44*^ (Fig. 2D). Western blot analysis (Fig. 4D) indicates an impairment of the myCAF phenotype (Fig. 4C), reflected in the complete loss of COL1A1 and a reduction of ɑSMA in CAF^*ΔCD44*^, supporting the hypothesis of CAF inactivation following *CD44* knockout. After TGFβ1 administration to the CD44-expressing CAFs, we observed higher COL1A1 protein levels (Fig. 4D). However, TGFβ1 did not lead to the re-expression of COL1A1 or the normalization of the ɑSMA level in CAF^*ΔCD44*^ cells indicating that the knockout of *CD44* disturbs their response to TGFβ1. Also, IL-6 did not lead to a change of protein expression in the CAF^*ΔCD44*^. Moreover, we observed in immunofluorescence analysis that the ɑSMA-containing stress fibers, which are present in CAFs, are absent in CAF^*ΔCD44*^ (Fig. S3C).

To investigate the influence of CD44 on the iCAF subtype, CAFs and CAF^*ΔCD44*^ were seeded in Matrigel (Fig. 4E), since stiff surfaces favor a myofibroblastic phenotype [37]. Downregulation of *COL1A1* and upregulation of *IL6* upon IL-1β induction confirmed a polarization into iCAFs. CAF^*ΔCD44*^ did not upregulate *IL6* indicating a disturbed response to IL-1β (Fig. 4E). TGFβ1-induced CAF^*ΔCD44*^ presented no *COL1A1* expression along with reduced *ACTA2* (ɑSMA), while both IL-1β and TGFβ1-induced CAF^*ΔCD44*^ showed low levels of *FAP*. Interestingly, as observed on culture plastic, CAF^*ΔCD44*^ remained round in Matrigel, while the CD44-expressing CAFs elongated (Fig. 4E). The findings indicate that the knockout of *CD44* in CAFs reverts their activation and renders them unresponsive to TGFβ1 and IL-1β.

### The secretome and immunosuppressive capacity of CAFs are altered after knockout of *CD44*

**Fig. 5:**
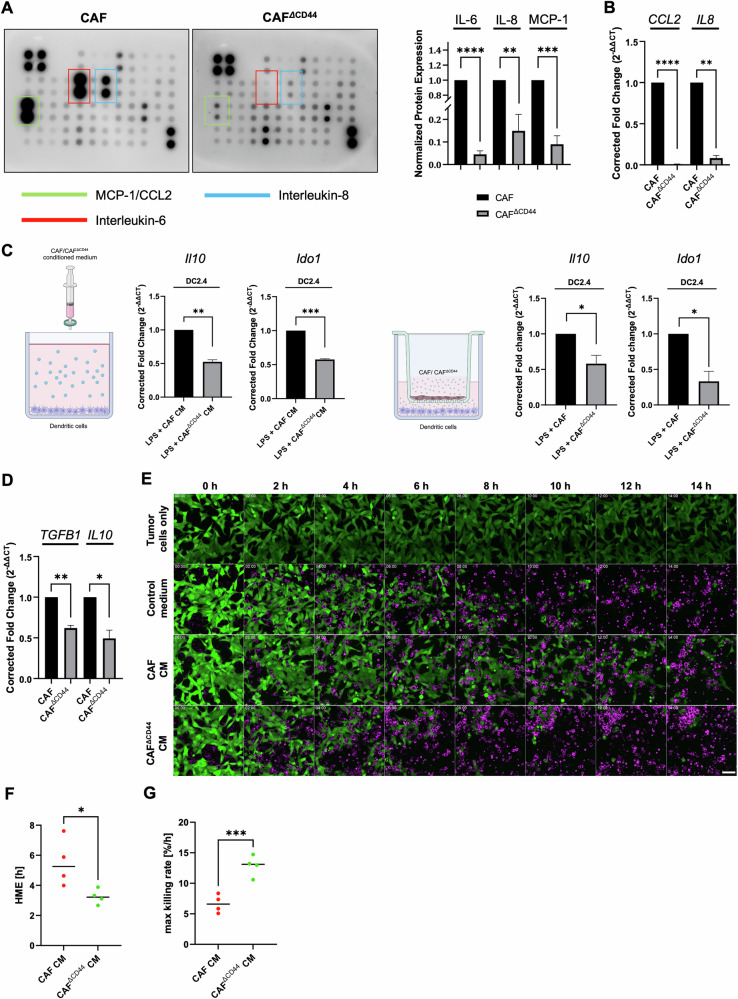
CAF^*ΔCD44*^ change their secretome and show a diminished immunosuppressive effect. **A** Conditioned media (CM) from CAFs and CAF^*ΔCD44*^ were collected and analyzed *via* cytokine array. Relevant proteins were highlighted by colored rectangles and subsequently quantified using ImageLab software. The mean intensity of the array spots was evaluated and normalized. The normalized protein expression of IL-6, IL-8, and MCP-1 is depicted as ratios comparing CAFs to CAF^*ΔCD44*^. *N* = 3. Data are means ± S.E. Statistical significance was determined using the one sample *t* test. **p value < 0.01; ***p value < 0.001; ****p value < 0.0001. **B** RNA of CAFs and CAF^*ΔCD44*^ was extracted 48 h after seeding and subjected to qPCR analysis of expression of *CCL2* and *IL8*. *GAPDH* was used as a reference gene. *N* = 3. Data are means ± S.E. Statistical significance was determined using the one sample *t* test. **p value < 0.01; ****p value < 0.0001. **C** DC2.4 were either cultured with 50% CAFs/CAF^*ΔCD44*^ conditioned media (CM) or indirectly co-cultured with CAFs/CAF^*ΔCD44*^ (as indicated in the illustrations: Treffert, S. (2026) https://BioRender.com/ifl9gho) for 48 h and treated with LPS. At 24 h after seeding, 1 µg/ml lipopolysaccharide (LPS) was added to the cultures. *Il10* and *Ido1* expression was assessed *via* qPCR. *Gapdh* and β*actin* were used as reference genes. Data are means ± S.E. Statistical significance was determined using the one sample *t* test. *N* = 3. *p value < 0.05; **p value < 0.01; ***p value < 0.001. **D** RNA of CAFs and CAF^*ΔCD44*^ was extracted 48 h after seeding and subjected to qPCR analysis of *TGFβ1* and *IL10* expression. *GAPDH* was used as a reference gene. Data are means ± S.E. Statistical significance was determined using the one sample *t* test. *N* = 3. *p value < 0.05; **p value < 0.01. **E** Fluorescence analysis of tumor cell confluency (CellTracker^TM^ Green) in different experimental conditions including or not human T cells (CD45-AF647, purple), CD3xPSMA BiTEs as well as control medium, CAF CM or CAF^*ΔCD44*^ CM. Imaging was conducted with the Cell Voyager CQ1 Confocal Quantitative Image Cytometer (Yokogawa) for 24 h in intervals of 30 min. Scale bar, 50 µm. **F** Fitted curves of tumor cell confluency were used to calculate the half-maximal effect (HME) of the two CM conditions. **G** The maximal killing rates at the HME of both conditions were calculated. Statistical significance was determined using a paired, two-tailed *t* test. *N* = 4, *n* = 2. *p value <0.05; ***p value < 0.001.

CAFs promote immune evasion by reshaping immune signaling, impairing T cell activation, and recruiting immunosuppressive cell populations through secreted factors [38]. In our study, secretome analyses of CAFs and CAF^*ΔCD44*^ revealed significant changes in a variety of secreted factors. The most apparent difference was observed in the secretion of IL-6, IL-8, and monocyte chemoattractant protein-1 (MCP-1) (Fig. 5A). Correspondingly, the gene expression of both *IL8* and *CCL2* (MCP-1) was markedly reduced (Fig. 5B), as observed for *IL6* (Fig. 4D).

CAFs can promote immunosuppression through the secretion of IL-6, which converts DCs into regulatory DCs and reduces T cell responses [14]. We therefore investigated how the secreted factors of CAFs and CAF^*ΔCD44*^ influence the expression of the immunosuppressive mediators IL-10 and indoleamine-2,3-dioxygenase-1 (IDO1) in the LPS-induced dendritic cell line DC2.4. The conditioned medium (CM) of CAFs upregulates *Il10* and *Ido1* expression in DC2.4 to a higher level compared to that of CAF^*ΔCD44*^ (Fig. 5C). The same results were observed in indirect transwell co-cultures of CAFs or CAF^*ΔCD44*^ with DC2.4.

CAF-derived factors have been shown to suppress T cell function directly [38]. Interestingly, two of the most potent immunosuppressive cytokines influencing T cells, TGFβ1 and IL-10, were significantly less expressed in CAF^*ΔCD44*^ (Fig. 5D). To determine whether CAF^*ΔCD44*^ affected CD8^+^ T cells differently compared to CD44-expressing CAFs, we assayed T cell cytotoxicity with the CQ1 confocal live-cell imaging system (Fig. 5E, Fig. S4A, Fig. S4B [Video]). CD3^+^ T cells from human peripheral blood mononuclear cells (PBMCs) were co-cultured with endogenously PSMA-expressing LNCAP cancer cells for 24 h while being incubated in the CM of CAFs and CAF^*ΔCD44*^ or control medium containing bi-specific T cell engagers (BiTEs) targeting CD3 and PSMA. CellTracker Green staining enabled us to assess tumor cell confluency in real time (Fig. S4C). Based on the measured confluency (Fig. S4C), the half-maximal effect (HME) was calculated (Fig. 5F). T cells incubated with the CM of CAFs were less efficient in killing the target cells, reflected in the prolonged time to reach the HME (5 h), contrary to T cells that were incubated in CAF^*ΔCD44*^ CM (<4 h). Additionally, the maximal killing rate at the HME was significantly higher in CAF^*ΔCD44*^ CM-treated T cells compared to CAF CM-treated T cells (Fig. 5G). These results show that CAFs lacking CD44 have a reduced ability to support the immunosuppressive function of immune cells of the tumor microenvironment.

### Knockout of *Cd44* in PDGFRβ^+^ cells in vivo reduces abundance of FOXP3^+^ T_reg_

**Fig. 6:**
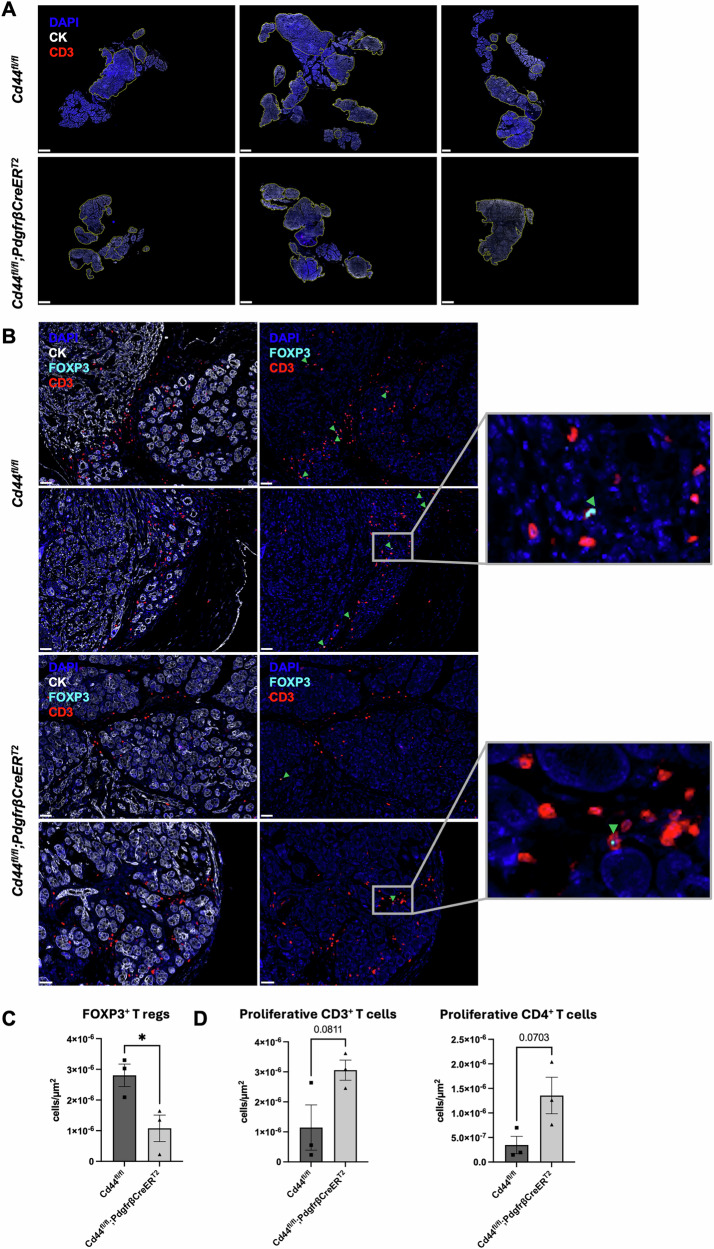
Knockout of *Cd44* in PDGFRβ^+^ cells in vivo reduces abundance of FOXP3^+^ T_reg_. **A** Multiplex IHC analysis tissue overview of the *Cd44*^*fl/fl*^*;PdgfrβCreER*^*T2*^ and control tumors excised two weeks after injection of FC1245 cancer cells and three weeks after i.p. injection of tamoxifen to knockout *Cd44* from PDGFRβ^+^ cells. Tumors were stained for DAPI (blue), cytokeratin (CK, white), and CD3 (red). Yellow lines delineate the area of the tissue with CK^+^ tumor cells. The magnification of the overview images was chosen to depict the entire tissue on the slide. Scale bar, 1 mm. **B** Exemplary areas of the *Cd44*^*fl/fl*^*;PdgfrβCreER*^*T2*^ and control tumors with cells double positive for CD3 (red) and FOXP3 (light blue). CD3^+^FOXP3^+^ cells are indicated with green arrows. Scale bar, 50 µm. **C** Quantification of CD3^+^FOXP3^+^ cells per µm^2^ on single-cell classification data generated by QuPath. Data are means ± S.E. Statistical significance was determined using the Student’s *t* test. *N* = 3. *p value < 0.05. **D** Quantification of the number of CD3^+^ki67^+^ (left graph) and CD4^+^ki67^+^ cells per µm^2^. Statistical significance was determined using the Student’s *t* test. *N* = 3.

In parallel to the in vitro analysis of CD44 function in immunosuppression, we analyzed the immune cell distribution in tumors from the experimental cohort described in Fig. 1 using multiplex immunofluorescence (Fig. 6A). Assessment of the intratumoral T cell compartment revealed a general low abundance of CD3^+^ T cells, consistent with an immunologically “cold” tumor microenvironment (data not shown). Nevertheless, a significant reduction in CD3^+^ FOXP3^+^ T_reg_ was observed in the cytokeratin (CK)-positive tumor area of *Cd44*^*fl/fl*^*;PdgfrβCreER*^*T2*^ mice (Fig. 6B, C). Accordingly, there was a tendency toward increased proliferation of CD3^+^ and CD4^+^ T cells in tumors with reduced T_reg_ abundance following *Cd44* deletion in *PDGFRβ*^*+*^ stromal cells (Fig. 6D). Collectively, these findings indicate that stromal *Cd44* expression contributes to the regulation of the intratumoral immune microenvironment. These immune changes are consistent with a reprogramming of CAF-associated stromal functions, suggesting that CD44-dependent CAF activity may contribute to immune suppression in PDAC.

## Discussion

CAFs contribute to key hallmarks of PDAC, including the extensive deposition of desmoplastic ECM and immunosuppression [39]. However, although therapeutic strategies targeting CAFs or their interactions with cancer or other stromal cells have shown anti-tumorigenic potential, complete CAF depletion can also enhance metastasis and reinforce immunosuppression [18, 34, 40], likely reflecting the functional heterogeneity of this stromal compartment. Besides the two most predominant subtypes, myCAFs and iCAFs, other distinct CAF subtypes have been described, including antigen-presenting CAFs (apCAFs [10]) and interferon-regulated CAFs (ifCAFs [41]). Nevertheless, myCAFs and iCAFs remain the two most prominent and best-characterized CAF subpopulations in PDAC [42].

In this study, we have shown that stromal CD44 is a central regulator of myCAFs and iCAFs, thus positioning it as a crucial molecular hub that integrates mechanical and immune signaling within the tumor microenvironment. We have demonstrated that CD44 expression in fibroblasts is required for their activation into contractile, matrix-producing, and cytokine-secreting CAFs, and that its loss reprograms these cells toward a non-activated phenotype.

Mechanistically, our findings indicate that CD44 acts as a co-receptor in TGFβ1 signaling, a key driver of CAF activation and desmoplasia [11]. CD44 enhances SMAD2 phosphorylation through its association with TGFβRI, a mechanism proposed to promote signaling in metastatic breast cancer cells [43]. There, the binding of HA to CD44 is indirectly involved in the phosphorylation of TGFβRI, while the link to CD44 does not directly influence the TGFβ1-mediated phosphorylation. This contrasts with our study in imPSCs, where CD44 deletion reduced TGFβ1-mediated Smad signaling, thereby preventing the full acquisition of the myCAF phenotype. Olsen and colleagues showed that TGFβ1 alone is not sufficient to fully promote myofibroblastic polarization in hepatic stellate cells [44]. Matrix stiffness and its sensing through the YAP/TAZ pathway play a crucial role in establishing a myofibroblastic feed forward loop. Activated fibroblasts also remodel and stiffen the matrix around them, ensuring their own sustained activation [45]. The accompanying changes in CAF morphology and their capability to contract collagen matrices, suggest that CD44 also contributes to this mechanotransduction, potentially by linking TGFβ1/Smad activity to the YAP/TAZ pathway, which integrates matrix stiffness and actomyosin tension [46–48].

Using a fibroblast-derived matrix system, we evaluated the topography of imPSC-derived matrices and discovered that upon TGFβ1-mediated activation, *Cd44*-depleted imPSCs produce isotropic matrices in contrast to *Cd44*-expressing imPSCs. Importantly, we showed fewer parallel fiber organization features that likely promote disease progression through stiffness-regulated mechanotransduction signaling [49]. In line with our model, Luong & Cukierman demonstrated that pharmacological modulation of TGFβ signaling can normalize pancreatic CAFs by altering ECM alignment and reducing pro-tumorigenic cytokine secretion [50]. In agreement with this, Alexander and colleagues demonstrated that the F-actin organizing protein palladin is upregulated in response to TGFβ1 and is indispensable for maintaining CAF activation, ECM alignment, and the secretion of immunosuppressive cytokines in PDAC [51]. This suggests that CD44 may act upstream of the TGFβ1-palladin axis, serving as a scaffold that enhances receptor signaling and thereby promoting actin cytoskeletal organization. The loss of CD44 could therefore dampen SMAD phosphorylation and palladin-dependent cytoskeletal remodeling, resulting in the isotropic and less inflammatory stroma.

We demonstrated that the iCAF phenotype is triggered by the exposure to IL-1β resulting in a high expression of cytokines like IL-6 [11] (Fig. 4E). In CD44-deficient CAFs, we showed that IL-1β does not induce the expression of the inflammatory CAF activation marker *IL6* or *FAP*, suggesting that CD44 is also involved in the IL-1β-mediated induction of the iCAF phenotype. Interestingly, in chondrocytes, knockdown of CD44 attenuated the IL-1β-mediated upregulation of IL-6, supporting the suggested role of CD44 in this pathway [52].

Besides IL-6, other key immunosuppressive cytokines like IL-8, CCL2, IL-10 and TGFβ1 are downregulated in CD44-deficient CAFs. In several cancer types, these factors affect the T cell-mediated anti-tumor response. IL-6, IL-8 and CCL2 were shown to affect the polarization of macrophages into tumor-promoting M2 TAMs [12], to drive the conversion of DCs into regulatory DCs [14] and to support MDSC differentiation and recruitment [13, 53], all suppressing a functional T cell response [38]. Consistent with these findings, our in vitro data show that CD44-deficient pancreatic CAFs have a reduced ability to induce a regulatory DC phenotype. Deletion of CD44 in CAFs relieved suppression of direct T cell-mediated tumor cell killing in vitro by significantly elevating the killing rate of T cells. This might result from the downregulation of CAF-derived TGFβ1 or IL-10 in CAFs that decrease essential cytolytic enzymes in CD8^+^ CTLs [54] or induce the expression of PD-L1 on tumor cells, thus limiting CD8^+^ T cell responses [55]. In vivo, stromal deletion of CD44 resulted in a decreased number of T_reg_ in the tumors that exhibited reduced primary tumor volume. This cell population dampens a functional T cell response by e.g. downregulation of T cell proliferation or stimulation of DCs to produce IDO1 [56] and is induced and maintained by two of the most prominent immunosuppressive cytokines TGFβ1 and IL-10 [57], reduced in CAFs lacking CD44. Furthermore, IDO1 was shown to sustain the T_reg_ phenotype, which was reduced alongside IL-10 in DCs that were exposed to CAFs lacking CD44 [58, 59]. Interestingly in melanoma mouse models, partial depletion of ɑSMA^+^ CAFs led to tumor regression, less abundance of T_reg_ and a higher cytotoxic potential of CD8^+^ T cells [60], comparable to our in vitro and in vivo data regarding CAFs inactivated by the knockout of *Cd44*. Hence, the blocking of T_reg_ recruitment and maintenance combined with the relief of suppression towards CD8^+^ T cells through CAF inactivation might have led to the immunological control of the tumor resulting in decreased tumor volume in the knockout animals.

The dual suppression of TGFβ1- and IL-1β-dependent programs implies that CD44 functions upstream of CAF subtype specification. These results provide direct evidence that fibrosis and immune evasion are mechanistically coupled through CD44.

Therapeutically, depletion of CD44 in CAFs offers a strategy to remodel the desmoplastic and immune-excluded landscape of PDAC into a more responsive and treatable state. In contrast to CAFs-depletion studies, our data present a nuanced strategy for functional reprogramming of CAFs to normalize rather than eliminate the stroma.

## Methods

### Mice

B6.Cg-Tg(Pdgfrb-cre/ERT2)6096Rha/J mice [26] were crossed with *C57BL/6-TgCd44-[exon3]*^*flox/flox*^ mice to generate the *Cd44*^*flox/flox*^*Pdgfrb-CreER*^*T2*^ (*Cd44*^*fl/fl*^*;PdgfrβCreER*^*T2*^) mouse line. The resulting line was used for PDGFRβ^+^ cell-specific *Cd44* knockout experiments. For in vivo purposes, only male mice (6 to 10 weeks old) were subjected to experiments. No randomization and no blinding were conducted. All animals were housed and maintained in facilities approved by the Regierungspräsidium Karlsruhe (Germany) under specific pathogen-free conditions and were handled according to EU directives for animal experimentation. The experiments were authorized by the Regierungspräsidium (35-9185.81/G-10/19) and termination criteria were approved.

### Cell lines

FC1245 cells were kindly gifted by Dr. Dave Tuveson (Cold Spring Harbor Laboratory, Cold Spring Harbor, NY, USA). HEK293T cells were obtained from ATCC (Wesel, Germany) and imPSCs were obtained from Associated Prof. Mathison and Prof. Urrutia (Medical College of Wisconsin, Milwaukee, WI, USA) [27]. These cell lines were cultured in DMEM containing GlutaMAX supplement (Gibco, Thermo Fisher Scientific, Waltham, MA, USA) and 10% fetal bovine serum (FBS, Gibco) as well as 1% Pen/Strep (P/S, Gibco). Human pancreatic CAFs were obtained from Vitro Biopharma (Denver, CO, USA) and were cultured in MSC-GRO™ Pancreatic CAF Maintenance Medium (Vitro Biopharma, Inc., Denver, CO, USA) with 1% P/S. DC2.4 cells were obtained from Merck (Darmstadt, Germany) and were cultured in RPMI-1640 (Gibco, 10% FBS, 1% GlutaMAX supplement, 10% 1 M HEPES, 0.1% β-mercaptoethanol, 1% non-essential amino acids, 1% P/S). Cell lines were tested regularly for mycoplasma contamination.

### Orthotopic PDAC cell implantation

Six- to ten-week-old *Cd44*^*fl/fl*^*;PdgfrβCreER*^*T2*^ or control mice were injected intraperitoneally with 20 mg/ml of Tamoxifen (Sigma-Aldrich, Merck, Darmstadt, Germany) dissolved in peanut oil, once per day for 5 days. At day seven, 4.5 × 10^4^/ 30 µl FC1245 cells in sterile PBS (Gibco) were injected into the pancreas of the mice. Animals were sacrificed 14 days after tumor cell inoculation, and tumors were excised and measured using an electronic caliper. The volume was calculated using the following formula: volume = length x width x height x 0.5 (mm^3^).

### Isolation of fibroblasts from the pancreas of *Cd44*^*fl/fl*^*;PdgfrβCreER*^*T2*^ mice

Fibroblasts from the pancreas of *Cd44*^*fl/fl*^*;PdgfrβCreER*^*T2*^ mice were isolated using a protocol modified from Waise and colleagues [61]. Mice were sacrificed and the pancreas was excised. Single-cell suspensions were established using the Miltenyi MACS^®^ mouse tumor dissociation kit (Miltenyi Biotec, Bergisch Gladbach, Germany) according to the manufacturer’s protocol. Single cells were collected by centrifugation and red blood cell lysis was performed using ammonium-chloride-potassium (ACK) buffer. After centrifugation, cells were resuspended in DMEM containing GlutaMAX supplement, 10% FBS and 1% P/S and seeded on culture plates. After attachment, the plates were washed three times with PBS before re-adding medium. After one week of culture, remaining adherent cells were identified as fibroblasts by testing for protein levels of ɑSMA and PDGFRβ *via* western blot analysis.

### 3D culture of CAFs

5 × 10^3^ CAFs or CAF^*ΔCD44*^ were seeded in 50 µl Matrigel (Corning, Corning, NY, USA) domes into 24-well plates and cultured in 500 µl of MSC-GRO™ pancreatic CAF maintenance medium (Vitro Biopharma, Inc.) with 1% of P/S. Retrieval of cells from the Matrigel was achieved by incubation with 250 µl of pre-warmed dispase (1 U/ml, Stemcell Technologies, Vancouver, BC, Canada). Collected cell pellets were prepared for further analysis.

### Cell culture

For immunofluorescence analyses, 1 × 10^4^ imPSCs/imPSC^*ΔCd44*^ or CAFs/CAF^*ΔCD44*^ per well were seeded on glass slides in 12-well plates. The cells were induced with 50 ng/ml recombinant TGFβ1 (PeproTech, Thermo Fisher Scientific, Waltham, MA, USA) in DMEM containing 1% FBS and 1% P/S for 24 h.

For induction experiments, cells were seeded in Matrigel, on flasks or plates. In 2D culture, CAFs/CAF^*ΔCD44*^ (1 × 10^5^) or imPSCs/imPSC^*ΔCd44*^ (9 × 10^4^) were seeded into 6-well plates. Cells were starved in DMEM containing 1% FBS and 1% Pen/Strep for 24 h, before cells were induced with 50 ng/ml of recombinant TGFβ1 (either 24 or 72 h) or 20 ng/ml of IL-1β or IL-6 (PeproTech). Cells were either prepared for protein or RNA analyses or removed from the Matrigel for RNA isolation.

### Lentivirus production and cell transduction

The CRISPR/Cas9-mediated knockout of *Cd44/CD44* in imPSCs and CAFs was performed using a third-generation lentiviral system. HEK293T packaging cells were transfected (PromoFectin, PromoCell, Heidelberg, Germany) with pMDL (5 µg*, gag* and *pol* genes), REV (2.5 µg, *rev* gene) and VSV-G (2.8 µg, *vsv-g* gene). The fourth plasmid (10 µg) either encoded a Cas9 endonuclease and a small guide RNA (sgRNA) to target the *Cd44* gene sequence (mouse: VB180221-1067cyf human:VB180221-1056peh) or a scramble sequence tagged with mCherry (VB240604-1056nya).

After six hours of transfection, the medium was removed, and 5.5 ml of growth medium were added into the culture plate. After 24 h of particle production, the medium was removed and filtered (0.45 µm) on top of the target cells. This was repeated once. The cells were selected with puromycin (2 µg/ml, InvivoGen, San Diego, CA, USA) and analyzed/sorted for CD44-negative or mCherry-positive cells (scramble) *via* flow cytometry.

### Flow cytometry

CRISPR/Cas9-transduced CAFs or imPSCs were collected, incubated with human (Clone FC1, BD Pharmingen^TM^, Franklin Lakes, NJ, USA; RRID: AB_2728082) or mouse Fc Block^TM^ (Purified rat anti-mouse CD16/CD32; BD Pharmingen^TM^; RRID: AB_394656) for 30 min and stained with a PE anti-mouse/human CD44 antibody (1:100, clone: IM7, BioLegend, San Diego, CA, USA; RRID: AB_312959) or isotype control (1:100, BioLegend; RRID: AB_326552) for 15 min. Cells were washed with FACS buffer (PBS + 2% FBS + 2 mM EDTA) and analyzed by flow cytometry using a FACSAria Fusion cytometer (BD Biosciences, Franklin Lakes, NJ, USA).

### Indirect immunofluorescence

Cells on coverslips were fixed with 2% paraformaldehyde and permeabilized with 0.1% (v/v) Triton-X-100. After blocking (5% FBS in PBS, 45 min, RT), samples were incubated with primary antibodies against CD44 (IM7, 2.5 µg/ml, BD Biosciences, RRID: AB_393732), COL1A1 (1:200, Cell Signaling, Danvers, MA, USA; RRID: AB_2800169), Phalloidin (1:1 000, Alexa Fluor 488, Thermo-Fisher Scientific), GFAP (1:200, Cell Signaling, RRID: AB_2631098) or ɑSMA (1:200, Cell Signaling, RRID: AB_2857972) diluted in 5% FBS in PBS overnight at 4 °C. After washing with PBS-Tween, cells were incubated with the secondary antibodies goat anti-rat IgG secondary antibody, Alexa Fluor® 488 conjugate 1:1 000; Thermo Fisher, RRID: AB_2534074, goat anti-rabbit IgG secondary antibody, Alexa Fluor® 546 conjugate (1:1 000; Thermo Fisher, RRID: AB_2534115) and 4’,6-dia-midino-2-phenylindole (DAPI) (1:1 000, Dako, Santa Clara, CA, USA) diluted in 5% FBS in PBS at RT for 30 min. Coverslips were mounted using Mowiol 4-88 (Roth, Karlsruhe, Germany). The slides were analyzed with a Zeiss LSM 800 confocal microscope (Zeiss, Oberkochen, Germany). Second harmonic generation signal was acquired at 430 nm by using linear unmixing mode after excitation at 860 nm.

### Bimolecular fluorescence complementation assay

TGFBRI-VN and CD44-VC were transfected using Fugene 4 K (Promega, Madison, WI, USA) into imPSCs. At 48 h after transfection, cells were fixed with 4% paraformaldehyde. Cells transfected with only TGFBRI-VN were used as negative controls. Cell nuclei were stained with DAPI for 15 min. Confocal images (Zeiss LSM 800) were processed using ImageJ software (National Institutes of Health, Bethesda, MD, USA). Sequences of the constructs can be provided on request.

### Co-cultures

#### Incubation of DC2.4 cells with CAFs/CAF^*ΔCD44*^-conditioned medium (CM)

2 × 10^5^ DC2.4 cells were seeded in a 6-well plate and incubated with full RPMI-1640 medium or with RPMI-1640 medium containing 50% of either CAF or CAF^*ΔCD44*^ CM. 24 h after seeding, 1 µg/ml LPS was added and incubated for 24 h before RNA was extracted for downstream analysis.

#### Indirect co-culture of CAFs/CAF^*ΔCD44*^ with DC2.4 cells

DC2.4 cells were seeded in a 12-well plate with 1 × 10^5^ cells per well. 5 × 10^4^ CAFs/CAF^*ΔCD44*^ were seeded in ThinCert inserts (0.4 μm pore, Greiner Bio-One, Kremsmünster, Austria) on top of the DC2.4. After 24 h of co-culture, 1 µg/ml of LPS was added to the well and incubated for 24 h, before RNA was extracted from the DC2.4 for downstream analysis.

### Protein detection

#### Human cytokine array

Secretome analyses of CAFs/CAF^*ΔCD44*^ were conducted using the Human Cytokine Antibody Array (Abcam, Cambridge, UK) according to the manufacturer’s instructions. 2 × 10^5^ CAFs/CAF^*ΔCD44*^ were seeded and incubated for 48 h. Media was collected, centrifuged (450 rcf, 6 min) and filtered (0.2 µm). Array membranes were incubated with CM and through a biotin-streptavidin-HRP antibody system, the factors present were visualized with the ChemiDoc™ Touch Imaging system (BioRad, Hercules, CA, USA) upon administration of the HRP substrate. Positive and negative control spots were used to quantify the relative signal intensity.

### Cell-derived matrices production

The procedure was adapted from Kaukonen et al., *Nat Protoc*, 2017, with minor modifications [32]. Plates with sterile coverslips were coated with 1% gelatin and crosslinked with 1% glutaraldehyde (v/v) for 20 min at RT. Crosslinking was quenched with 1 M glycine for 20 min at RT, followed by PBS washes. Cells were seeded at 4 × 10^5^ per well in DMEM (10% FBS, 1% P/S). After 48 h, extracellular matrix deposition was stimulated by daily treatment with 1% ascorbic acid (50 μg/ml) with or without TGFβ1 (5 ng/ml) for 7 days. Fibroblasts were then removed using extraction buffer (100 mM NH_4_OH in PBS containing 0.5% (v/v) Triton X-100) for 3 min, followed by two PBS washes. Cell-derived matrices were stored at 4°C in PBS or fixed with 4% paraformaldehyde for 10 min prior to fluorescent staining and confocal microscopy.

### Contraction assay

The contraction assay was adapted from Chitty et al., *Cancer Reports*, 2020, with minor modifications [62]. 96-well plates were coated with 2% BSA and 2.5 × 10⁴ cells were embedded in 100 µL of rat tail collagen I hydrogel (1.5 mg/mL, Corning, Corning, NY, USA). Hydrogels were polymerized for 1 h at 37 °C, after which 100 µL of complete growth medium was added to each well. Gels were allowed to contract for 48 h at 37 °C. Images of the collagen lattices were acquired using a MICA microscope (Leica, Wetzlar, Germany), and the diameter of the well and gel was measured using ImageJ.

### T cell killing assay

CD3^+^ T cells were isolated from human PBMCs using the Pan T cell isolation kit (Miltenyi Biotec, 130-096-535) according to the manufacturer’s protocol. T cells were activated for three days using CD3/CD28 activation beads (Dynabeads^TM^ Human T-activator CD3/CD28, Gibco) at a 1:1 bead-to-cell ratio and 30 U/mL recombinant IL-2 (R&D Systems, Minneapolis, MN, USA; BT-002-050) in serum-free X-VIVO 15 medium (Lonza, Basel, Switzerland). Tumor cells were seeded 20 h prior the experiment on fibronectin-coated 96-well imaging plates (Screenstar Microplate, Greiner Bio-One; fibronectin: R&D Systems, Minneapolis, MN, USA, 1030-FN-05M). On the day of co-culture, tumor cells were stained with CellTracker^TM^ Green CMFDA (Invitrogen^TM^, Thermo Fisher). T cells and target cells were suspended in the experimental (60% CAF conditioned media and 40% RPMI-1640 (HiGlutaXL RPMI-1640, HiMedia, AL028G) + 10% FBS (Bio & Sell, Feucht, Germany; FBS.S0615)) and control media containing Anti-CD45-AF647 (1:500, Invitrogen^TM^, Thermo Fisher, MA5-38730) for labeling T cells and NucSpot 568/580 (1:2 000, biotium, Fremont, CA, USA 41036) for identifying dead cells. The CD3xPSMA BiTEs (MedChemExpress, Monmouth Junction, NJ, USA; HY-P99802) were prepared in the respective experimental and control medium and added to the co-culture at 10 µg/ml. Imaging was conducted with the Cell Voyager CQ1 Confocal Quantitative Image Cytometer (Yokogawa, Tokyo, Japan) for 24 h at intervals of 30 min. Maximum intensity projection images were recorded from three Z planes covering a 3 µm using a 20x/0.8 NA objective (Olympus, Tokyo, Japan; UPLXAPO20X).

The tumor cell confluency was measured based on the green CellTracker fluorescence using CellPathfinder (version 3.06.01.08, Yokogawa). The confluency over time was baseline-corrected and depicted as percent difference [100*(Value-Baseline)/Baseline].

The resulting curves were fitted using the [Inhibitor] vs. response - Variable slope four parameter logistic models [Y=Bottom + (Top-Bottom)/(1 + (IC50/X)^HillSlope)]. To calculate the maximal killing rate, the first derivative at t=half-maximal effect (HME) was calculated using:

$${{\rm{Killing\; Rate}}}_{\max }={\left|\frac{{dY}}{{dX}}\right|}_{X={HME}}=\left|\frac{\left({\rm{Top}}-{\rm{Bottom}}\right)\,{\rm{HillSlope}}}{4\,{HME}}\right|$$

### Opal multiplex immunohistochemistry (mIHC)

#### Staining and image acquisition

Multiplex immunofluorescence staining was performed on 3 µm FFPE tissue sections using the Opal™ workflow (Akoya Biosciences, Marlborough, MA, USA) on a Leica BOND RX automated platform (Leica Biosystems, Nussloch, Germany). Sections were baked at 40 °C for 15 min, followed by 58 °C for 1 h, then deparaffinized. Heat-induced epitope retrieval was performed in BOND Epitope Retrieval Solution 2 (ER2, pH 9; Leica Biosystems) for 40 min. Endogenous peroxidase activity was quenched with 3% H₂O₂ (Sigma-Aldrich) for 10 min, and non-specific binding was blocked with BOND Protein Block (Leica Biosystems) for 10 min at room temperature. Sequential staining involved incubation with primary antibodies (see Supplementary Table) for 30 min, followed by HRP-conjugated secondary antibodies (Akoya Biosciences) and Opal tyramide signal amplification (1:150–1:250 dilution, 10 min; Akoya Biosciences). High-stringency washes were applied between cycles, and heat-induced epitope retrieval (ER2, 20 min) was performed between markers to remove bound antibodies while preserving fluorophores. Two panels were designed: Panel 1 five markers and Panel 2 containing two markers (see Supplementary Table). Nuclei were counterstained with DAPI, slides were coverslipped with Aquapoly Mountant (Polysciences), and multispectral images were acquired on the Akoya PhenoImager HT system (Akoya Biosciences). Image unmixing was performed using inForm software (Akoya Biosciences), with fluorophore assignments optimized to minimize spectral overlap.

#### Image analysis

Image analysis was performed using QuPath (Version 0.6.0; Bankhead, P., Loughrey, M.B., Fernández, J.A. et al. 2017). Whole tissue annotation and segmentation were based on DAPI signal. Cell detection was performed using the built-in cell detection command in QuPath. The detection parameters were customized and optimized to achieve accurate segmentation of the whole tissue. For each marker, object classifiers were independently developed using a representative training image compiled from all samples to avoid bias. Classifiers were based on characteristic features such as signal intensity patterns, subcellular localization, and morphological criteria. Subsequently, the trained classifiers were applied sequentially to each segmented sample. The export of single-cell classification data from QuPath was conducted in Excel format for subsequent analysis. The resulting data were then plotted in GraphPad Prism 10, where the *t* test, Mann–Whitney-U test, and Outlier test were used to compare cell densities between genotypes.

### Single cell data analysis

Raw read counts were obtained from the Genome Sequence Archive (GSA; CRA001160). Data processing and quality control were performed using Scanpy. Low-quality cells were filtered out according to the following criteria: only cells expressing between 100 and 6000 genes and with fewer than 100,000 total counts were retained. Genes detected in fewer than three cells were excluded. Doublet detection was performed using the scanpy.pp.scrublet function, identifying 0.26% of cells as potential doublets, which were excluded from downstream analyses. After filtering, 45,776 cells remained for further analysis.

Read counts were normalized per cell to a total count of 10,000. Principal component analysis (PCA) was performed on the 5000 most variable genes, followed by nearest-neighbor graph construction using the top 30 principal components. UMAP was applied for dimensionality reduction and visualization. Cell type annotations from the original publication were used as the reference labels. Marker gene analysis was conducted using the Wilcoxon rank-sum test, comparing each cell type against all others. Comparisons between pancreatic cancer and normal conditions were performed using the same approach. Genes with an adjusted *p*-value below 0.05 were considered as significantly regulated. Fibroblastic cells were defined by the expression of ɑSMA, PDGFRβ, lumican (LUM), decorin (DCN), adipogenesis regulatory factor (ADIRF), COL1A1.

### Statistical analysis

Tests for Gaussian (normal) distribution were conducted for all data by Shapiro-Wilk normality test or Kolmogorov-Smirnov normality test. Upon passing the normality test, both samples were assumed to derive from populations with the same variances. Using a non-paired parametric test like the one-sided unpaired Student’s *t* test, mean values of quantitative variables between two independent groups were compared. In case of comparison between the mean of a single sample against a hypothetical mean a one-sample *t* test was used. If means of more than two samples were compared, a one-way analysis of variances (ANOVA) was performed and Holm-Šídák’s multiple comparison was used as a post hoc test. Data are shown as the average with ± standard error of mean (SEM). We accepted a significance level α < 0.05. For animal experiments the statistical power (1-β) was 0.8 while ɑ was 0.05. *P* values are indicated by asterisks and defined as *p = 0.05, **p = 0.01, ***p = 0.001, ****p < 0.0001. Statistical analysis was performed using GraphPad Prism 9.3.1 software (GraphPad, RRID:SCR_002798).

Supplementary information is available at Cell Death & Disease’s website.

## Supplementary information

Supplementary Figure S1

Supplementary Figure S2

Supplementary Figure S3

Supplementary Figure S4

Video for Supplementary Figure S4

Video for Supplementary Figure S4

Supplementary Data and Methods

Western Blot raw data

## Data Availability

The data that support the findings of this study derive from Peng et al. [24] and are available in the Genome Sequence Archive under project PRJCA001063. The accession number for the sequencing data is GSA: CRA001160.
