# Supplementary material for: Loss of CD44 re-educates pancreatic cancer-associated fibroblasts modulating their fibrotic and immunosuppressive functions
Source: Cell Death Dis. 2026 Aug 15;17(1):729. doi: 10.1038/s41419-026-09155-5 (PMC13477481; doi:10.1038/s41419-026-09155-5)
Supplement: Supplementary file 1 [file 41419_2026_9155_MOESM7_ESM.docx]

**Supplementary Figure legends:**

**Supplementary Figure 1 | Validation of primary fibroblastic cells from *Cd44^fl/fl^;PdgfrβCreER^T2^* mice and *Cd44* knockout in imPSC cell line. A** Cells were isolated from the pancreas of *Cd44^fl/fl^;PdgfrβCreER^T2^* mice by enzymatic and mechanical dissociation and cultured. Cells were evaluated for the expression of the fibroblast markers PDGFRβ and ɑSMA *via* western blot. **B** Flow cytometry analysis of the CD44 expression in imPSCs after CRISPR/Cas9-mediated knockout. imPSCs and imPSC*^ΔCD44^* cells were stained with panCD44 antibodies (IM7) coupled to phycoerythrin (PE) and the corresponding rat IgG2b, κ isotype control, also labelled with PE.

**Supplementary Figure 2 | *Cd44* knockout and siRNA-mediated reduction in imPSCs influence activation and EMT. A** imPSCs were transfected with control siRNA (*Ctrl*siRNA) or siRNA targeting all CD44 isoforms (*Cd44*siRNA) and induced with recombinant TGFβ1 for 24 hours. Transfected imPSCs were analyzed *via* IF using specific antibodies against CD44 (IM7, AlexaFluor546, red) and COL1A1 (AlexaFluor488, green). Nuclei were counterstained with DAPI (blue). Scale bar, 50 µm. The CTCF of all conditions was determined by quantifying the fluorescence *via* ImageJ with deduction of background signals. qPCR analysis was used to determine the expression of *Cd44* and *Col1a1*. *Gapdh* and *βactin* were used as reference genes. **B** imPSCs and imPSC*^ΔCD44^* were induced with recombinant TGFβ1 (50 ng/ml) or IL-1β (20 ng/ml) for 24 hours. *Has1, Has2 and Has3* gene expression was analyzed by qPCR. *Gapdh* and *βactin* were used as reference genes. N=3. Data are means ± S.E. **C** Co-immunoprecipitation (Co-IP) of CD44 and TGFβRI in imPSCs. Whole cell lysates were incubated with a CD44 antibody (clone: KM201) or IgG control (rat IgG1, κ) and precipitated. CD44 and TGFβRI were detected *via* western blot. Input represents non-precipitated lysate of TGFβ1-treated cells. **D** Expression analysis of *Snai1*, *Twist1*, *Zeb1* and *EpCam* via qPCR after TGFβ1 treatment. *Gapdh* and *βactin* served as reference genes. **E** Chromobody (CB) plasmids encoding the Alpaca-derived vimentin-specific V_H_H-eGFP fusion protein (VB6-eGFP) were transfected into imPSCs and imPSC*^ΔCD44^* target cells. Representative confocal images show chromobodies binding to endogenous vimentin (green). Scale bar, 50 µm. eGFP intensity upon TGFβ1 stimulation for 72 hours was quantified *via* flow cytometry in imPSCs and imPSC*^ΔCD44^*. Data are means ± S.E. Statistical significance of all experiments depicted here was determined using the one-sided unpaired Student’s t-test. N=3. *p-value<0.05, **p-value<0.01, ***p-value<0.001.

**Supplementary Figure 3 | Generation of CRISPR/Cas9-edited human CAF cell line and corresponding scramble cell line. A** Flow cytometry analysis of the CD44 expression in CAFs after CRISPR/Cas9-mediated knockout after sorting. CAFs and CAF*^ΔCD44^* cells were stained with CD44 antibodies (IM7) coupled to PE and the corresponding rat IgG2b, κ isotype control (PE). **B** Flow cytometry analysis of mCherry signal in CAFs and CAFs transduced with a CRISPR scramble construct marked with mCherry. Brightfield picture of CAF transduced with the sgRNA-mCherry. Scale bar, 100 µm. **C** IF analysis of CAFs and CAF*^ΔCD44^* using specific antibodies against CD44 (IM7, AlexaFluor488, green) and ɑSMA (AlexaFluor546, orange). Nuclei were counterstained with DAPI (blue). Scale bar, 50 µm. N=1.

**Supplementary Figure 4** | **T cell-mediated tumor cell killing assay in various experimental conditions and corresponding quantifications. A** Overview IF pictures of tumor cell confluency (CellTracker^TM^ Green) and human T cells (CD45-AF647, purple) in all experimental conditions (tumor cells only, control medium, CAF CM or CAF*^ΔCD44^* CM). Images were created in a time span of 24 hours with the Cell Voyager CQ1 Confocal Quantitative Image Cytometer (Yokogawa) at intervals of 30 minutes. **B** Corresponding videos of the CAF CM and CAF*^ΔCD44^* CM-incubated conditions showing T-cell mediated tumor cell killing. **C** The tumor cell confluency was measured based on the green CellTracker fluorescence using CellPathfinder. The confluency over time was baseline-corrected and depicted as percent difference [100*(Value-Baseline)/Baseline] and the curves were fitted. N=4, n=2.

**Supplementary methods:**

***Vimentin-Chromobodies***

VB6-eGFP-Chromobody expression vectors were kindly provided by Prof. Ulrich Rothbauer (Eberhard Karls University, Tübingen, Germany). imPSC and imPSC*^ΔCD44^* were transfected with the expression vector encoding the VB6-eGFP-Chromobody (CB) sequence [31] using Lipofectamine 3000 (Thermo-Fisher Scientific) according to the manufacturer’s protocol. 24 hours post transfection, cells were selected using 0.5 mg/ml G418 for 72 hours. Vimentin expression was induced in selected cells using 50 ng/ml TGFβ1 for 72 hours and eGFP-positive cells were sorted using a FACSAria Fusion (BD Biosciences). For EMT induction experiments, 9x10^4^ cells were seeded into 6-well plates and treated with 50 ng/ml TGFβ1 in DMEM with 1% FBS and 1% Pen/Strep for 72 hours. Single cell suspensions were analyzed for GFP-positive cells using a FACSAria Fusion (BD Biosciences).

***Cd44 knockdown using siRNA***

9x10^4^ imPSCs were seeded into 6-well plates. 100 pmol of *Cd44*-specific siRNA or non-silencing control siRNA were diluted in 100 µl of DMEM (without FBS) per well and incubated with 12 µl of HiPerFect (Qiagen) for 7.5 min at RT. The complexes were added dropwise onto the cells and incubated. After 24 hours, a second transfection was performed, and cells were seeded for the respective experiments after 48 hours of total transfection. The sequences will be provided upon request.

***Protein isolation and detection***

Cells were lysed with 1% Triton lysis buffer (1% Triton-X-100; 150 mM NaCl; 50 mM Tris-HCl (pH 7.4); 5 mM Na_3_VO_4_; 25 mM NaF; 0.1% NP-40; 1 mM EDTA; 1 mM EGTA; 50X protein inhibitor cocktail (Roche); adjust pH to 7.0). Lysates were collected and centrifuged (13 200 rpm, 20 min, 4 °C), followed by the transfer of the supernatant to fresh tubes. 4x Laemmli sample buffer (BioRad) with 10% β-mercaptoethanol was added, and samples were boiled at 95 °C for 5 min.

Co-Immunoprecipitation (Co-IP)

imPSCs were lysed using 1% Triton lysis buffer. 50 µl of the supernatant was taken as input control and mixed with 4x Laemmli sample buffer (BioRad) with 10% β-mercaptoethanol, heated at 99°C for 5 minutes and stored at -20°C. The remaining supernatant was used for immunoprecipitation. To target the protein of interest and its interacting partners, samples were incubated overnight with the panCD44 (clone: KM201) antibody on an orbital shaker at 4°C. On the following day, Agarose G beads were washed three times with 1% Triton lysis buffer followed by centrifugation at 1200 rpm for 3 minutes (pre-clearing). Subsequently 30 µl of the beads were added to the samples. For the IgG control, directly coupled beads were used. The samples were incubated for 3h at 4°C on an orbital shaker. After the incubation period, samples were washed three time and centrifuged (1 200 rpm, 3 min, 4°C) to spin down the beads coupled to the antibodies and the proteins. The supernatant was removed, and the beads were resuspended in 1% Triton lysis buffer and washed three times for 5 minutes at 4°C on an orbital shaker. After another centrifugation (1 200 rpm, 3 min, 4°C), the supernatant was completely aspirated and the proteins were denatured in 2x Laemmli sample buffer (BioRad) with 10% β-mercaptoethanol by boiling at 99°C for 5 minutes.

Western Blot

SDS-PAGE was conducted using 5% stacking and 8% separating gels to separate proteins. Electrophoresis ran for 60 min at 100 V and for 45 min at 120 V. Transfer of proteins onto PVDF membranes was executed with the Trans-Blot Turbo system (Bio-Rad). After blocking of the membranes with 5% BSA in TBS-T for 1 h, they were incubated overnight at 4 °C with primary antibodies (1:200 - 1:1 000 in 5% BSA/TBS-T). Membranes were washed three times with TBS-T, before being incubated 1h at room temperature with HRP-conjugated secondary antibodies (1:2 000 in TBS-T). After three washing steps, ECL substrate was applied and HRP-mediated signals were visualized with the ChemiDoc Touch Imaging System (Bio-Rad).

***RNA extraction, reverse transcription and qPCR***

Total RNA was extracted using the RNeasy Mini Kit (Qiagen). For the generation of cDNA from freshly isolated mRNA the qScriber^TM^ cDNA Synthesis (highQu) was used according to the manufacturer’s protocol. The cDNA was then diluted in a 1:4 ratio in RNase free water. The qPCR reactions (20 µl) consisted of 10 µl 2x GoTaq® qPCR Master Mix (Promega), 0.5 mM reverse and forward primers, and 2 µl cDNA. The qPCR was performed on the CFX96 Touch Real-Time PCR detection system (Biorad). *Gapdh* and *βactin* were used as reference genes and the relative quantification was calculated based on the Litvak method (ΔΔCt). Primers were designed using the Primer3 open software (http://primer3.ut.ee) using transcript sequences from the NCBI-database.

**Supplementary Table:**

Supplementary Table 1: Antibody panels used in the opal multiplex immunohistochemistry analysis.

| Staining round | Primary antibody | Opal dye |
| --- | --- | --- |
| Panel 1 | | |
| 1 | Cytokeratin (Agilent DAKO, GA053, 1:1 in PBS) | Opal 480 (Akoya, OP-001000, 1:150) |
| 2 | FOXP3 (CST, 12653S, 1:1000) | Opal 520 (Akoya, OP-001001, 1:250) |
| 3 | CD4 (Sino Bio, 50134-R001, 1:2000) | Opal 570 (Akoya, OP-001003, 1:250) |
| 4 | CD3 (CST, 78588, 1:500) | Opal 620 (Akoya, OP-001004, 1:150) |
| 5 | Ki67 (Invitrogen, MA5-14520, 1:500) | Opal 690 (Akoya, OP-001006, 1:150) |
| Panel 2 | | |
| 1 | CD8 (CST, 98941, 1:500) | Opal 480 (Akoya, OP-001000, 1:150) |
| 2 | CD3 (CST, 78588, 1:500) | Opal 690 (Akoya, OP-001006, 1:150) |
