# Supplementary material for: Loss of CD44 re-educates pancreatic cancer-associated fibroblasts modulating their fibrotic and immunosuppressive functions
Source: Cell Death Dis. 2026 Aug 15;17(1):729. doi: 10.1038/s41419-026-09155-5 (PMC13477481; doi:10.1038/s41419-026-09155-5)
Supplement: Supplementary file 2 [file 41419_2026_9155_MOESM8_ESM.docx]

**Western blot raw data:**

**Figure S1A:**

*PDGFRβ:*

*ɑSMA:*

*Β-Actin:*

**Figure 2C:**

*CD44:*

*COL1A1:*

*Vinculin:*

**Figure 2E:**

*pSMad2:*

*Smad2:*

**Figure S2C:**

*Co-IP:*

Input - TGFß1

+ TGFß1

- TGFβ1

IgG

Input + TGFß1

+ TGFß1

- TGFβ1

IgG

**Figure 4D:**

*CD44:*

*ɑSMA:*

*COL1A1:*

*Vinculin:*
